# Repetitive transcranial magnetic stimulation regulates neuroinflammation in neuropathic pain

**DOI:** 10.3389/fimmu.2023.1172293

**Published:** 2023-04-25

**Authors:** Yi-Wen Bai, Qi-Hao Yang, Pei-Jie Chen, Xue-Qiang Wang

**Affiliations:** ^1^ Department of Sport Rehabilitation, Shanghai University of Sport, Shanghai, China; ^2^ School of Rehabilitation Science, Shanghai University of Traditional Chinese Medicine, Shanghai, China; ^3^ Department of Rehabilitation Medicine, Shanghai Shangti Orthopaedic Hospital, Shanghai, China

**Keywords:** neuropathic pain, repetitive transcranial magnetic stimulation, neuroinflammation, analgesic effect, analgesic mechanism

## Abstract

Neuropathic pain (NP) is a frequent condition caused by a lesion in, or disease of, the central or peripheral somatosensory nervous system and is associated with excessive inflammation in the central and peripheral nervous systems. Repetitive transcranial magnetic stimulation (rTMS) is a supplementary treatment for NP. In clinical research, rTMS of 5–10 Hz is widely placed in the primary motor cortex (M1) area, mostly at 80%–90% RMT, and 5–10 treatment sessions could produce an optimal analgesic effect. The degree of pain relief increases greatly when stimulation duration is greater than 10 days. Analgesia induced by rTMS appears to be related to reestablishing the neuroinflammation system. This article discussed the influences of rTMS on the nervous system inflammatory responses, including the brain, spinal cord, dorsal root ganglia (DRG), and peripheral nerve involved in the maintenance and exacerbation of NP. rTMS has shown an anti-inflammation effect by decreasing pro-inflammatory cytokines, including IL-1β, IL-6, and TNF-α, and increasing anti-inflammatory cytokines, including IL-10 and BDNF, in cortical and subcortical tissues. In addition, rTMS reduces the expression of glutamate receptors (mGluR5 and NMDAR2B) and microglia and astrocyte markers (Iba1 and GFAP). Furthermore, rTMS decreases nNOS expression in ipsilateral DRGs and peripheral nerve metabolism and regulates neuroinflammation.

## 1 Introduction

Neuropathic pain (NP) is a common chronic pain condition that substantially influence patients’ quality of life and is caused by a disease or lesion affecting the somatosensory nervous system (1, 2). According to epidemiological research, the frequency of NP can be as high as 7% to 8% in the general population, accounting for 20% to 25% of those who experience chronic pain (3, 4). The basic characteristics of NP include sensory abnormalities, spontaneous pain, and hyperalgesia (5). NP management focuses on treating symptoms. In addition to using recommended first-line antidepressants and antiepileptic drugs, repetitive transcranial magnetic stimulation (rTMS) has demonstrated effectiveness in subjects who have not responded to conventional medical treatment (1, 6).

The central and peripheral nervous systems exhibit excessive inflammation in neuropathic pain, which may contribute in initiating and maintaining chronic pain. NP state is associated with infiltrating various immune cells near or within peripheral nerves. Macrophages, cytokines, T-lymphocytes, and chemokines are key players in neuroinflammation and initially used to promote healing and regeneration (7). At the early stage of NP, macrophages and neutrophils sensitize sensory neurons of the dorsal root ganglia (DRG) *via* mediators such as tumor necrosis factor-alpha (TNF-α) and diverse interleukins(ILs), such as interleukin-10 (IL-10), interleukin-1β (IL-1β), interleukin-4 (IL-4) and interleukin-17 (IL-17), and interferon-γ (8–11). Sensitized afferents activate microglia by releasing ATP, colony stimulating factor 1 (CSF1), chemokines (CCL2, CX3CL1), and proteases, resulting in up-regulation of microglial markers such as ionized calcium-binding adapter molecule 1 (Iba-1) and CD11b in the spinal cord (8, 12, 13). According to a recent explosion in microglial research, numerous signaling molecules are changed in microglia and play a key role in the pathophysiology of NP (14). Microglia proliferation and activation elicit astrocytes, which release pro-inflammatory agents and promote neuronal activity. In addition to microglia and astrocytes, the primary afferent neurons and bone marrow-derived invading and tissue-resident macrophages cooperate to regulate pain signals in peripheral tissues (15). Molecular and cellular imaging of NP helps locate nerve injury or neuroinflammation with accuracy and confidence (16). MicroRNAs and other noncoding RNAs are recently explored as potential master switches that could connect nerve damage, pain, and inflammation (7). In a recently published systematic review and meta-analysis of 38 studies, the outcome demonstrated that rTMS has analgesic effects on NP. According to a subgroup analysis, the key determinants of treatment efficacy include treatment site, stimulation frequency, and interaction between frequency and lesion of the stimulation site (17).

Studies on the mechanisms of rTMS in NP are ongoing. rTMS-induced analgesia is based on the remodeling of the endogenous opioid system and the restoration of normal cortical excitability (18, 19). rTMS also produces analgesic effects by inhibiting the transmission of nociceptive signals (20, 21). The activated motor cortex induced by rTMS may alter the activity of the nearby motor cortex and the thalamic nuclei. Increasing studies have been conducted on how rTMS interact with the nociceptive system by inducing anti-inflammatory cytokines. This review discussed the interrelationships between rTMS and neuroinflammation of NP models and patients.

## 2 Effect of rTMS on neuropathic pain

European rTMS has wide clinical applications and continuously distributes multiple pulses at a fixed frequency compared with chronic implant therapy (22). The clinical application of rTMS in managing NP involves many central and peripheral nervous diseases, including stroke, Parkinson’s disease, spinal cord injury, and sciatica (23). Guidelines on neuromodulation for chronic pain published in the European Academy of Neurology provided a weak recommendation for using rTMS targeting the primary motor cortex (M1) in NP (24). Low-frequency (1 Hz) rTMS can inhibit nerve cell metabolism and reduce cortex excitability, and high-frequency (≥1 Hz) rTMS can boost nerve cell metabolism and cerebral cortex’s excitability (25). The International Union of Clinical Neurophysiology guideline states that high-frequency rTMS stimulation of the contralateral M1 area has a definite analgesic effect on NP, and a long and continuous course of treatment is highly beneficial and recommended for Grade A (26, 27). In addition to M1, patients with severe pain or depression may benefit from treatment in the left dorsolateral prefrontal cortex (PFC) (28). The concentrated stimulation site of rTMS, such as the “8” coil, is superior to the circular coil and has high stimulation frequency, large stimulation pulse, and many stimulation times, making it an effective method to improve pain. Stimulating the M1 area near the representative pain area can produce a potent analgesic effect (29, 30).

Hasan (31) used 10 Hz rTMS for five sessions as an intervention for patients with PSP to explore the effect of rTMS on pain sensitivity and somatic sensory function of patients with NP. The results showed that the affected body’s sensory defect to temperature change was significantly improved, and the pain score was greatly relieved, indicating that rTMS might improve the neural pathway shared by noxious stimulus and temperature signals. Concerning the persistence of the therapeutic effect of NP caused by refractory SCI managed by rTMS, Yilmaz et al. (32) adopted a 10 Hz rTMS treatment program for 10 consecutive days and followed up with the subjects for 10 days, 6 weeks, and 6 months to evaluate their pain. The results showed that rTMS could relieve pain for up to 6 weeks. Hosomi et al. (33) conducted four weekly sessions of 5 Hz rTMS in the hand area of M1 patients with upper extremity NP and the foot region of lower extremity pain, and the subjects showed significant analgesic effects after the treatment cycle. However, Hodaj H et al. (34) applied 10Hz rTMS to stimulate the motor cortex of 57 patients with chronic NP in different regions for 12 daily sessions in 3 weeks, and all the patients experienced significant pain relief compared with baseline. A guideline on the safety, ethical considerations, and clinical application of rTMS revealed that 20 Hz stimulation for the treatment of central NP could also provide effective pain relief by stimulating the M1 area on the opposite side of the patient’s pain area (35). Jin Y et al. (36) found regardless of the intensity (5, 10, or 20 Hz), rTMS had an effect on the relief of chronic NP and the pain could be relieved to the maximum extent after the fifth stimulus. High-frequency rTMS therapy for NP is mostly more than 80% of the resting motor threshold, and the total pulse number is more than 1000 (37). rTMS therapy is usually administered once a day for 10–15 consecutive days. The pain relief degree of patients with a stimulation duration greater than 10 days is higher than that of patients with a stimulation duration less than 10 days. The shorter the treatment duration, the shorter the duration of the analgesic effect (38). Currently, no explicit therapeutic parameter of rTMS for NP caused by different diseases has been established in the academic circle. The main site of rTMS is the M1 region for stimulation, and high-frequency stimulation at above 5 Hz is mainly used for analgesia. More research information about the effect of rTMS on NP is shown in **Table 1**. High-frequency rTMS has demonstrated a good therapeutic effect on NP, and the stimulation site is often on the contrary side of the pain cortex for central stimulation. In clinical practice, 5–10 Hz rTMS is frequently applied in the M1 area contralaterally of NP, typically at an RMT of 80%–90%; 5–10 treatments are usually sufficient to produce a noticeable analgesic effect (39).

**Table 1:** Major findings of rTMS in neuropathic pain studies.

Author, year	Study type	Subjects	Sample size	rTMS Parameters	Outcome measures	Analgesiceffect
Hosomi et al., 2013 (105)	Cross-over	Patients with PSP	57 Gender not 60.7±10.6	Coil type: F-8 Stimulation site: M1 Frequency: 5 Hz Intensity: 90%RMT Duration: 10 sessions	VAS	Modest pain relief
Matsumura et al., 2013 (106)	Cross-over	Patients with PSP	20 12M,8F 63.6±8.1	Coil type: F-8 Stimulation site: M1 Frequency: 5 Hz Intensity: 100%RMT Duration: 1 day	VAS	Pain relief
Hasan et al., 2014 (31)	Case series	Patients with PSP	14 10M,4F 57(median)	Coil type: F-8 Stimulation site: M1 Frequency: 10 Hz Intensity: 80-90%RMT Duration: 5 sessions	NRS	Modest but significant pain relief
Yılmaz et al., 2014 (32)	Randomized control	Patients with SCI	32 16F, 16M 38.6±6.5	Coil type: F-8 Stimulation site: motor cortex Frequency: 10 Hz Intensity: 110%RMT Duration: 10 days	VAS	Middle-term (over 6 weeks) pain relief
Kobayashi et al., 2015 (107)	Cross-over	Patients with PSP	18 12M,6F 63.0±9.9	Coil type: F-8 Stimulation site: M1 Frequency: 5 Hz Intensity: 90%RMT Duration: 12 weeks	VAS	Pain relief
Sun et al., 2019 (108)	Randomized control	Patients with SCI	17 15M,2F 23.0-54.5	Coil type: F-8 Stimulation site: M1 Frequency: 10 Hz Intensity: 80%RMT Duration: 6 weeks	NRS	More pain relief from 2 weeks to 6 weeks
Hodaj et al., 2020 (34)	Randomized control	Patients with chronic NP	57 21M,36F 62.2±15.0	Coil type: F-8 Stimulation site: motor cortex Frequency: 10 Hz Intensity: 80%RMT Duration: 3 weeks + 5 months	VNS NPSI	All pain measures significantly decreased from baseline to the end of the treatment
Hosomi, K et al., 2020 (33)	Randomized control	Patients with intractable NP	142 93M,49F EG: 63.0±10.0 CG: 60.8±12.1	Coil type: F-8 Stimulation site: M1 Frequency: 5 Hz Intensity: 90%RMT Duration: 1 week + 1 month	VAS SF-MPQ2	Significant pain relief after the treatment cycle
Zhao et al., 2021 (109)	Randomized control	Patients with PSP	38 21M,7F 50.1±11.34	Coil type: F-8 Stimulation site: motor cortex Frequency: 10 Hz Intensity: 80%RMT Duration: 3 weeks	NRS MPQ	Significant pain relief
Kim, J. Y. et al., 2013 (70)	Animal study	Rats with SCI	10 F 203-250g	Coil type: Round Frequency: 25 Hz Intensity: 0.2 T Duration: 8 weeks	GFAP and Iba1	The expressions of Iba1 and GFAP elevated in dorsal and ventral horns
Yang, L. et al., 2018 (83)	Animal study	Rats with CPNP	32 M 180-250g	Coil type not Frequency: 20 Hz Intensity: 90% RMT Duration: 10 days	nNOS, GFAP, BrdU	The expression of nNOS, GFAP, BrdU decreased
Toledo, R. S. et al., 2021 (49)	Animal study	Rats with CCI	106 M 60 days old	Coil type: Bufferfly Frequency: 1 Hz Intensity: 200 mT Duration: 8 days	TNF-α and IL-10	The levels of IL10 and TNF-α increased in the PFC and spinal cord
Toledo, R. S. et al., 2021 (56)	Animal study	Rats with CCI	81 M 60 days old	Coil type: Bufferfly Frequency not Intensity not Duration: 8 days	BDNF, IL-10 levels and the object recognition test	rTMS reversed the impairment LTM and the increase in the hippocampal IL-10 levels
Hu, Y. et al., 2022 (60)	Animal study	Rats with CCI	32 M 8 weeks old	Coil type: Circular Frequency: 10 Hz Intensity: 1.5T Duration: 4 weeks	mGluR5, NMDAR2B, TNF-α, IL-1β and IL-6	The expressions of mGluR5, NMDAR2B, TNF-α, IL-1β and IL-6 reduced

PSP, post-stroke pain; SCI, spinal cord injury; CCI, chronic constriction injury; NP, neuropathic pain; CPNP, chronic peripheral neuropathic pain; M, male; F, female; EG, experimental group; CG, control group; rTMS, repetitive transcranial magnetic stimulation; Hz, hertz; F-8, figure eight; RMT, resting motor threshold; mT, millitesla, T tesla; M1, primary motor cortex; VAS, visual analogue scale; NRS, numerical rating scale; LTM, long term memory; MPQ, Mcgill pain questionnaire; VNS, visual numerical scale; NPSI, Neuropathic Pain Symptom Inventory; SF-MPQ2, short-form McGill pain questionnaire-2; GFAP, anti-glial fibrillary acidic protein; nNOS, neuronal nitric oxide synthase; BrdU, 5-bromo-2-deoxyuridine; TNF-α, tumor necrosis factor-alpha; IL, interleukin; PFC, prefrontal cortex; mGluR5, metabotropic glutamate receptors 5; NMDAR2B, N-Methyl-D-Aspartic acid receptor type 2B.

## 3 rTMS regulates neuroinflammation in neuropathic pain

**Figure 1:**
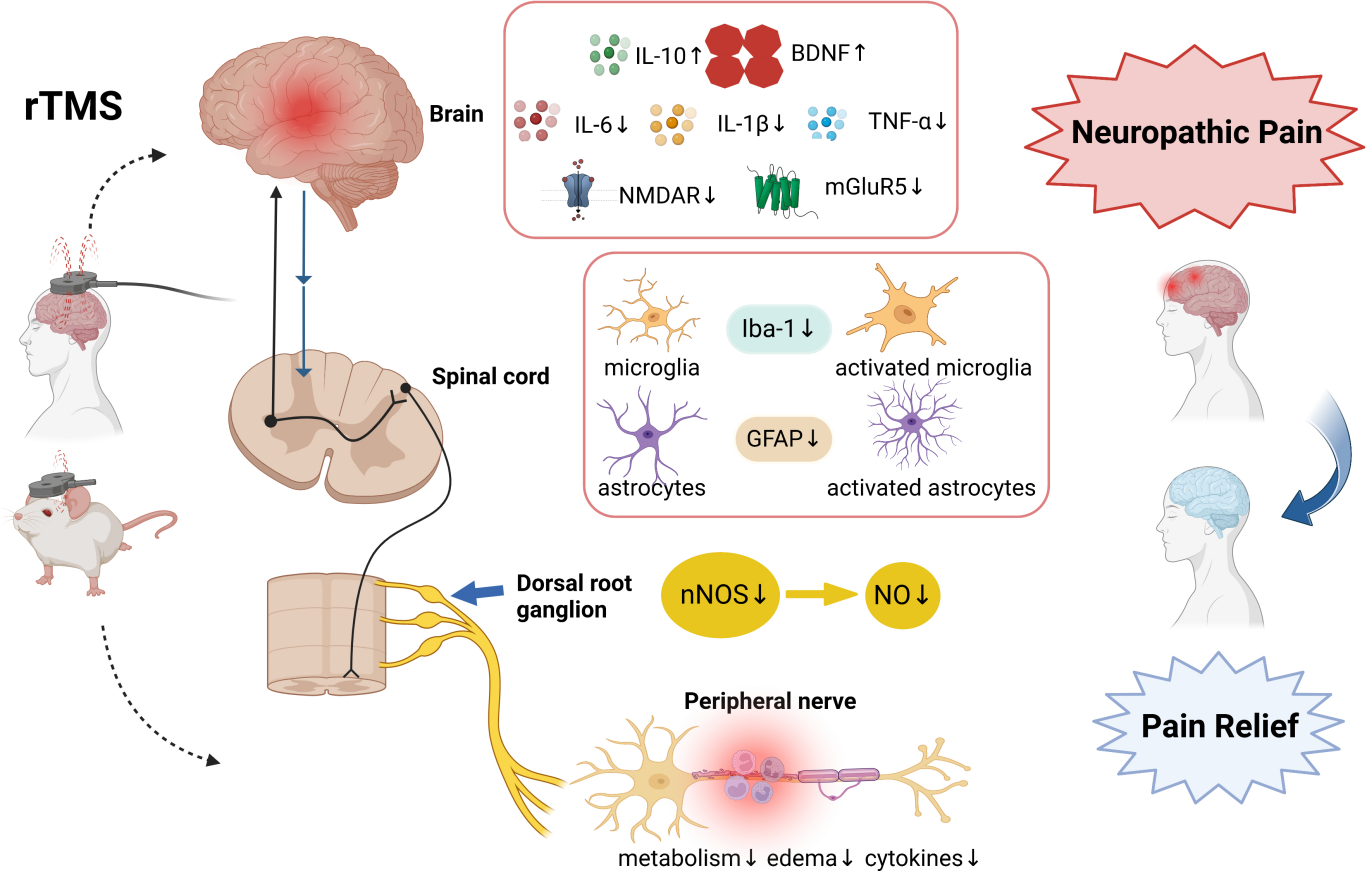
The involved mechanisms in rTMS on neuropathic pain relief. The analgesic mechanism involves neuroinflammatory n cytokines, glutamate receptors, microglia and astrocyte markers, nNOS, and peripheral nerve inflammation. Created with BioRender.com. rTMS, Repetitive Transcranial magnetic stimulation; IL-10, interleukin -10; IL-6, interleukin -6; IL-1β, interleukin -1β; BDNF, brain-derived neurotrophic factor; TNF-α, tumour necrosis factor-alpha; mGluR5, metabotropic glutamate receptors 5; NMDAR2B, N-Methyl-D-Aspartic acid receptor type 2B; Iba-1, Ionized calcium-binding adapter molecule 1; GFAP, glial fibrillary acidic protein nNOS, nitric oxide synthase; NO, Nitric Oxide.

Infections in the CNS rarely occur. The majority of infections and tissue damage occur at the periphery, and neuroinflammatory responses are triggered by peripheral inflammation, which involves the neurons, glia, and blood brain barrier. This neuroinflammatory response leads to synaptic dysfunction, neuronal damage, and worsening of several pathologies in the brain, spinal cord, dorsal root ganglia, and peripheral nerve (40–43). Immune cells can produce pro- or anti-nociceptive mediators, modulating pain in various ways. Pro-inflammatory cytokines such as TNF-α, interleukin-6 (IL-6) and IL-1β are mediators of inflammatory and neuropathic pain (44). Anti-inflammatory cytokines such as IL-10 and IL-4 act to regulate the inflammatory process, limiting tissue damage and restoring homeostasis (45). The interactions between inflammation and NP are bidirectional. These cytokines can adjust the inhibitory and excitatory synaptic transmission, ultimately improving pain signals’ transmission to the brain. rTMS increases the expression of anti-inflammatory and decreases that of pro-inflammatory cytokines (46–48), indicating that it plays a significant role in neuroinflammation (49) (see **Figure1**).

### 3.1 Effects of rTMS on cortical neuroinflammation

NP is associated with excessive cortical inflammation, leading to persistent pain (50). More severe brain inflammation can be observed in patients with NP compared with that in pain-free patients. The inflamed brain can induce oxidative stress and the release of pro-inflammatory factors, such as IL-1β, TNF-α, and nitric oxide (NO) (51, 52). The pain-related symptoms can be relieved by reducing neuroinflammation activation in the cortical areas and vice versa. Inflammatory changes in different brain regions are different. According to a study, NP decreases the complexity of excitatory synaptic connections and brain-derived neurotrophic factor (BDNF) expression in the hippocampus, and the opposite trends are dependent on TNF-α (53). BDNF is down-regulated in the PFC after NP (54). In the anterior cingulate cortex, which is the first response region to painful stimuli, NP increases the expression of TNF-α, chemokine CX3CL1, and IL-6 (55).

rTMS focuses on a particular cortical site to reduce neuroinflammation and has an analgesic effect. Toledo et al. (49, 56) demonstrated that 1 Hz rTMS treatment for 8 days could alter IL-10 levels in the hippocampus and PFC in NP rats. IL-10 plays a significant role in NP conditions. IL-10 reverses memory and learning deficits in NP models by preventing the negative effects of IL-1β or lipopolysaccharide on long-term potentiation (57–59). IL-6 and TNF-α dose-dependently induce the release of IL-10 by microglia. Hu et al. (60) found that rTMS at 10 Hz could noticeably reduce inflammatory factors in NP rats, including TNF-α, lL-6, and IL-1β in anterior agranular insular (AId). In NP rats, IL-1β levels rose in the brain’s prefrontal cortex, brainstem, hippocampus, and peripheral areas (61, 62). IL-1β and TNF-αprimarily mediate the neuroinflammation and stress response. Hu et al. also found that the left AId of the animals with NP caused by CCI exhibited rising levels of metabotropic glutamate receptors 5 (mGluR5) and N-methyl-D-aspartic acid receptor type 2B (NMDAR2B), and 10 Hz-rTMS intervention applied on the left AId could reverse the expression. NMDAR and mGluR5 are key receptors for pain processing and mental changes (63, 64). When NP is present, pro-inflammatory substances such as TNF-α and IL-1β enhance NMDAR-mediated current frequency and spontaneous postsynaptic current, stimulate glutamatergic neurotransmission, and result in mechanical hypersensitivity (65, 66). In addition, rTMS-treated NP rats presented highs level of BDNF in the PFC and hippocampus (49, 56). BDNF is a crucial neuromodulator in the nervous system with alternating persistent pain states and inflammation conditions (54). It is also related to nociceptive hypersensitivity. According to a report, rTMS improves the signaling between BDNF and its receptor TrkB in the cortex (67). rTMS alters the activation of cortical and subcortical structures, such as orbitofrontal cortices, anterior cingulate, medial thalamus, and periaqueductal gray matter (68). Overall, rTMS treatment has an essential impact on the nociceptive responses of NP and a beneficial effect on the levels of neurotrophin and anti-neuroinflammatory cytokines.

### 3.2 Effects of rTMS on spinal cord neuroinflammation

NP induces sustained inflammatory reactions in the spinal cord and NP rats to increase the level of cytokines, including IL-1β and TNF-α, and the activation level of microglia/macrophages in the dorsal horn (69). rTMS exhibits an inhibition effect on spinal cord inflammation, mainly related to the augmentation of anti-nociceptive effect in NP rats. Kim et al. (70)presented that rTMS applied on the motor cortex reduced the expression of Iba-1 and glial fibrillary acidic protein (GFAP) in spinal dorsal and ventral horns at the L4–L5 levels in NP rats (70). Pro-inflammatory cytokines, chemokines, and their receptors are constantly upregulated by activated microglia and astrocytes, resulting in chronic allodynia development (71–76). In another animal study, Luo et al. (77) stated that rTMS stimulation could control inflammation, suppress apoptosis, and promote neurogenesis. rTMS promotes the anti-inflammatory polarization of microglia. Through rTMS stimulation on microglia, anti-inflammatory cytokine production is promoted *in vivo* and *in vitro*. In general, rTMS can stimulate neurogenesis, enhance brain function recovery, and modify microglia polarization toward an anti‐inflammatory phenotype.

In addition to microglia activation and leukocyte infiltration, reactive astrogliosis is a crucial component of inflammation. High-frequency rTMS (50 Hz) can stimulate IL-6 release, boost Ca2+ inflow, and encourage astrocyte proliferation *in vitro* (78–80). In the spinal cord injury model, rTMS promoted reactive white matter astrocytes’ migration to a CNS lesion (81). In rats with regional brain and spinal cord injury, high-frequency rTMS could reduce glial activation and related neuroinflammation (70, 82). These findings suggested that astrocytic activation may be influenced by rTMS to decrease neuroinflammation. Yang et al. (83) also found that GFAP and 5-bromo-2-deoxyuridine (BrdU) in the dorsal horn were reduced after 20 Hz rTMS treatment for 10 days on M1 in NP rats induced by sciatic nerve ligation. GFAP and BrdU are often used to observe astrocyte proliferation and activation. Inflammation-associated GFAP abnormalities and astrocytic plasticity problems also impact neural tissue growth and repair, degeneration, and death. Overall, rTMS reverses mechanical hyperalgesia, reduces pain *via* suppressing the activations of astrocytes and microglia, and decreases pro-inflammatory cytokine staining in the NP model, especially central nerve pain after SCI.

### 3.3 Effects of rTMS on dorsal root ganglia neuroinflammation

NP is closely associated with the hyperresponsiveness of sensory neurons from DRG (84). Glial cells in the DRG produce inflammatory markers and contribute to the development of NP. Neurotrophic factors change NP inflammation in DRG neurons, activate inflammatory agents, and induce the expression of different ion channels (85–87). Inflammation drives molecular alterations in the DRG where IL-1β and purinoceptors are upregulated (88). TNF-α elevates phosphorylation (pERK) in small and medium-sized DRG neurons with NP (89). Yang et al. (83) presented that 20 Hz high frequency rTMS could attenuate brush-evoked and spontaneous pain symptoms and reduce neuronal nitric oxide synthase (nNOS) expression in ipsilateral DRGs in rats with peripheral NP. NO is an extremely unstable free radical gas related to spinal cord hyperalgesia and neurosensitization (90–92). A crucial enzyme in NO production is nitric oxide synthase (NOS). NOS isoform inhibition has recently been promoted as a potential treatment for NP conditions (93–95). Accordingly, rTMS alleviates NP conditions by suppressing nNOS overexpression in ipsilateral DRGs.

### 3.4 Effects of rTMS on peripheral nerve neuroinflammation

NP can be produced by inflamed but otherwise uninjured axons (96). Elevated levels of local and serum TNF-α, IL-6, IL-1β, and IL-2 are associated with neuroinflammation and peripheral inflammation in NP (7, 61). Molecules responsible for anti-inflammation have been linked to NP recovery, highlighting the association between inflammation and pain (97–99). “Remote effect” is the term used to describe how rTMS may impact the area of the brain related to the stimulation location and the stimulation site itself (100, 101). rTMS reduces sciatic nerve metabolism and raises the sciatic nerve function index in CCI rats. Increased spontaneous activity and metabolic alterations following nerve damage contribute to the symptoms of NP. Glucose metabolism is necessary for neuronal function, and PET imaging can identify areas with high metabolic activity using modified glucose molecules (16, 102). Following nerve damage, inflammation lowers the amount of glucose in the injured nerve tissue, elevating the phosphorylation of FDG and the relative SUV (103). In one study, the degenerative alterations of the sciatic nerve tissue were less severe in the rTMS groups than in the CCI group. Under light microscope observation, the 10 Hz rTMS treatment group presented complete and distinct sciatic nerve tissues. The inflammatory cells invaded the spaces between the nerve fibers, and the lymphocytes’ nuclei were rounded and heavily stained. In the 1 Hz rTMS-treated group, sciatic nerve pathological alterations were considerably more severe than those in the 10 Hz-rTMS group. Therefore, neuromodulation *via* rTMS may provide an accessible, practical, low-cost, and noninvasive approach to alleviate symptoms associated with NP (104).

## 4 Conclusion

This review aimed to summarize the neuroinflammation changes in NP induced by rTMS. In clinical research, rTMS of 5–10 Hz is widely applied in the primary motor cortex (M1) area, mostly at 80%–90% RMT, and 5–10 treatment sessions could produce an optimal analgesic effect. rTMS shows the anti-inflammation effect by decreasing pro-inflammatory cytokines, including TNF-α, IL-1β, and IL-6, and increasing anti-inflammatory cytokines, including IL-10 and BDNF, in cortical and subcortical tissue. rTMS also reduces mGluR5 and NMDAR2B expression levels, regulates neuroinflammation, down-regulates microglia and astrocytes markers Iba1 and GFAP, and decreases the expression of nNOS in DRGs and ipsilateral and peripheral nerve metabolism. In the future, additional *in vitro* and *in vivo* studies and clinical trials are required to investigate the mechanism of rTMS on NP and establish the foundation for the clinical treatment.

## Author contributions

X-QW conceived the review. Y-WB and Q-HY drafted the manuscript and searched the literature to identify eligible trials. Y-WB and Q-HY extracted and analyzed data. X-QW and P-JC revised the tables in the drafted manuscript. All authors contributed to the article and approved the submitted version.
